# Fipronil and Fipronil Sulfone Distribution in Chicken Feathers and Eggs after Oral and Dermal Exposure

**DOI:** 10.3390/foods10123077

**Published:** 2021-12-10

**Authors:** Francesco Corrias, Alessandro Atzei, Riccardo Taddeo, Nicola Arru, Mattia Casula, Rachid Salghi, Mariateresa Russo, Alberto Angioni

**Affiliations:** 1 Department of Life and Environmental Science, University of Cagliari, 09126 Cagliari, Italy; francesco.corrias@unica.it (F.C.); alessandro.atzei@unica.it (A.A.); ricky.taddeo@hotmail.it (R.T.); nicola.arru.logica@gmail.com (N.A.); mattiacasula92@gmail.com (M.C.); 2 National School of Applied Science, Ibn Zohr University, Agadir 80000, Morocco; r.salghi@uiz.ac.ma; 3 Department of Agricultural Science, Mediterranean University of Reggio Calabria, 89124 Reggio Calabria, Italy; mariateresa.russo@unirc.it

**Keywords:** hens, fipronil, fipronil sulfone, oral exposure, dermal exposure, LC-MS/MS, food control, food safety

## Abstract

This work aimed to investigate the bio-distribution and the persistence of fipronil and its primary metabolite fipronil sulfone after oral and dermal administration by simulating natural farming conditions. Fipronil and fipronil sulfone detection and quantification were performed in different poultry matrices using an LC-MS/MS method coupled with modified QuEChERS extraction. After oral administration, fipronil was detected in feathers at each sampling time, in eggs for 28 days, and in the internal organs at the end of the experiment. After dermal administration, high levels of fipronil were detected in feathers, accounting for 195.85 ± 8.54 mg/kg, which were reduced by a third after 60 days. No traces of fipronil were detected in the eggs or internal organs. In addition, fipronil sulfone showed remarkable residues in all samples in trial 2. The data obtained confirmed that inappropriate use of unauthorized pesticides can lead to severe contamination of entire poultry farms. The contemporary presence of fipronil sulfone in feathers and eggs associated with the lack of fipronil in eggs suggests recent dermal contamination or past oral contamination. Moreover, simultaneous analysis of hens’ feathers and eggs could represent a new method to improve large-scale monitoring programs and animal welfare, limiting their slaughter.

## 1. Introduction

Fluocyanobenpyrazole, better known as fipronil, is an insecticide of the phenylpyrazole family developed by Bayer Crop Science in 1987. Fipronil acts by contact or ingestion as an agonist at the gamma-aminobutyric acid (GABA) receptors, leading to the disruption of the central nervous systems of insects [1]. Fipronil shows broad-spectrum activity and sensitivity against ants, beetles, cockroaches, fleas, ticks, termites, mole crickets, thrips, rootworms, and weevils; and the parasites resistant to organic phosphorus, organic chlorine, pyrethroids, and carbamates [2]. Moreover, it has no cross-resistance with the pesticides already available in the market.

Since fipronil’s introduction, its metabolism, degradation, and bioremediation strategies have been primarily studied in animals and the environment [3,4]. Fipronil can undergo three metabolic pathways: photolysis leading to fipronil-desulfinyl, fipronil-sulfide reduction, and fipronil-sulfone oxidation [5]. Fipronil degradation is slow on crops and relatively slow in soil and water, depending on the substrate and external conditions [2]. 

On the contrary, after entering the body of a human or animal, fipronil is extensively oxidized to fipronil sulfone in the liver and distributed in tissues and organs [6]. No data are available regarding the degradation process of fipronil on feathers. The toxicity of fipronil is extremely variable and dependent on the affected species and dose; Adhikari et al. (2014) reported a decrease in blood parameters in white leghorn cockerels after fipronil oral administration, resulting in anemia and leukocyte suppression [7]. However, fipronil sulfone is more persistent and toxic to birds and other organisms than the parent compound [2]. 

In humans, fipronil has adverse effects on the liver, thyroid gland, and kidneys following long-term exposure [2]. In 2013, considering its severe toxicity and high acute risk represented for honeybees, the European Commission (EC) has forbidden its utilization on crops in the EU, though it is still allowed to treat domestic pets against parasites [5]. In addition, fipronil use is banned for controlling pests on animals intended for food use, such as chickens. 

This compound gained substantial media attention after its detection in chicken eggs collected in Belgium in 2017. Fipronil, improperly used in chicken farms, entered the food chain in the Netherlands rapidly becoming a major public health problem and involving several countries in Europe and Asia [8]. Residues of fipronil and its metabolite have been found in many monitoring trials on hens worldwide [9,10,11], leading to the seizure and destruction of eggs and the sacrificing of contaminated poultry [12]. 

The main reason for which fipronil is thought to be related to poultry products is its fraudulent application in laying hens for the treatment of the red mite *Dermanyssus gallinae*, a harmful chicken parasite [13]. Fipronil contamination can occur by topical application or through the administration of contaminated feed. 

Furthermore, bird preening activity can increase cross-contamination among the hens of poultry farms. The European Food Safety Authority (EFSA) defined fipronil residue as the sum of fipronil and its primary metabolite fipronil sulfone, expressed as fipronil [14].

The maximum residue level (MRL), the acceptable daily intake (ADI), and the acute reference dose (ARfD) in eggs and poultry products have been set to 0.005 mg/kg, 0.0002 mg/kg body weight (BW), and 0.009 mg/kg BW, respectively [14,15]. Several studies were conducted to detect fipronil and fipronil sulfone residues in food matrices, such as apple, cucumber, honey, cabbage, meat, eggs, and poultry [16,17,18,19,20,21], using different GC-MS and LC-MS/MS methods coupled with different extraction and partition techniques [22]. 

No studies dealing with the assessment of fipronil and fipronil sulfone bio-distribution in chickens after dermal or oral exposure were found prior to our study. Canton et al. (2020) performed both oral and dermal contamination trials with fipronil on laying hens; however, they evaluated the residues of fipronil and fipronil sulfone in eggs, excluding the muscle, internal organs, and feathers. Moreover, the amount of pesticide administered and the methods of dermal application were different [13]. Gerletti et al. (2020) presented a toxicokinetic model to predict the transfer of fipronil and fipronil sulfone from contaminated feed to the eggs of laying hens [23]. 

This work aimed to determine the amounts of fipronil and fipronil sulfone residues in eggs, feathers, muscle, and internal organs of laying hens and roosters to establish their distribution path after oral dermal contamination. In addition, a chronic and acute dietary risk assessment evaluation on breast muscle and eggs was performed. Two different trials simulating inappropriate feed contamination and an insecticide dermal application scenario were performed on laying hens and roosters. 

Oral contamination occurred through a suitable amount of polluted feed, whereas dermal application was achieved by spraying an aerosol of commercial fipronil solution. Following exposure, eggs and feathers were sampled at different times; at the end of the trials, animals were sacrificed, and the muscle and internal organs were collected and analyzed. 

Fipronil and fipronil sulfone detection and quantification were performed using an LC-MS/MS method validated to analyze 116 pesticides coupled with modified QuEChERS extraction.

## 2. Materials and Methods

### 2.1. Standard and Reagents

All analytical standards (purity 99.5–99.7%) were purchased from Dr Ehrenstorfer (Lab Service Analytica, Milan, Italy). Acetonitrile (ACN), formic acid, ammonium formate (≥99% purity), and methanol were LC/MS solvents (Sigma Aldrich, Milan, Italy). Double-deionized water with a resistivity less than 18.2 Ω was obtained with a Milli-Q purification system (Millipore, Bedford, MA, USA). The stock standard solution of the active ingredients (a.i.s) and a multi-standard mixed working solution (1000 mg/L) were prepared in acetonitrile and stored at 4 °C. Working standard solutions were prepared daily from the mixed working solution by dilution with the eluent mixture. 

QuEChERS (Quick, Easy, Cheap, Effective, Rugged, and Safe) reagents were catalog numbers 5982–6650, 4 g MgSO4, 1 g NaCl, 1 g trisodium citrate dihydrate, 0.5 g disodium hydrogen citrate sesquihydrate (En Method 15662, Agilent Technologies, Milan, Italy), catalog numbers 5982–5056, 150 mg PSA, and 900 mg MgSO4 (EN Method, Fruit, and Vegetable, Agilent Technologies, Milan, Italy). In addition, the fipronil spray solution at 2500 mg/L was purchased from a local pet shop.

### 2.2. Formulate Composition

Several aqueous formulations of fipronil are commercially available for application on animals and breeding environments. Therefore, six different commercial products (identified with the letters from A to E) were analyzed before starting the trials to confirm fipronil concentration and active ingredient integrity. Each formulation was appropriately diluted before LC-MS/MS analysis.

### 2.3. Animals Selection and Wellness 

Fifteen hens of 15 months and approximately 2 kg weight, each producing one egg/day, and five roosters of 2 months with an average weight of 1.4 ± 0.56 kg, in good physical condition, were provided by an egg-producing factory. Animals were divided into different groups and maintained in separate cages. The cages were placed in a stable environment at 25 °C and 50% relative humidity (RH) and were cleaned daily to avoid chicken contamination from feces. Trials were conducted during September–November 2012. 

The number of individuals was suitable for this work. A qualified internal committee analyzed the project set up; all animal trials and sacrifice techniques agreed with the ARRIVE guidelines and the EU Directive 2010/63/EU for animal experiments.

### 2.4. Study Design

Animals, acclimatized for one week, were supplied with food and water ad libitum. Oral and dermal contamination trials were performed using a 2500 mg/L fipronil commercial solution recommended for treating the parasites on dogs and cats. Five untreated hens (*n* = 5) were used as control and housed in individual cages. Animals were subjected to the following experimental procedures. 

Trial 1: Laying hens (*n* = 5) were exposed to chronic fipronil oral administration for one week by contaminated feed recommended for poultry (final fipronil and fipronil sulfone concentration: 25 mg/kg; about 100 g feed /day per hen) prepared by spraying the commercial fipronil solution on a single batch followed by vigorous agitation. The contaminated feed was replaced with unpolluted feed on the seventh day.

Trial 2: Laying hens (*n* = 5) and roosters (*n* = 5) were contaminated by a direct one-shot feather air spraying administration using the fipronil solution. The indications reported on the commercial product label suggest an application rate of 3–6 mL of solution per kg of body weight. In our experiment the entire animal body was sprayed with 25 mL of a fipronil solution at 10–20 cm distance; the contamination process was considered completed when the plumage of the animals was completely wet.

Samples of hen eggs (1 egg/hen/sampling; *n* = 5 per sampling) were collected at days 0, 1, 2, and 7, and subsequently every seven days in both trials, for a total of 115 eggs analyzed. In addition, the feathers of hens and roosters (15 feathers/hen or rooster/sampling; *n* = 75 per sampling) were collected on days 7, 14, 42, and 66 in trial 1; and days 1, 7, 49, and 66 in trial 2 for a total of 600 feathers collected. The trials lasted 66 days. At the end of the study, all hens and roosters were sacrificed; breast and internal organs were dissected, weighed, and homogenized before residue analysis.

### 2.5. Non-Compliant Eggs

Different batches of seized non-compliant eggs ready for the market, for a total of 80 eggs, were collected from farms subjected to confiscation by the authorities and analyzed.

### 2.6. Sample Processing and Extraction

A 5.0 g sample of homogenized fresh eggs, 5.0 g of poached eggs, 0.5 g of feathers, or 1 g of liver, breast, brain, heart, kidney, or thigh with skin was weighted in a 50 mL test tube and extracted according to Anastasiades et al. (2003), with some modifications [24]. Briefly, 10 mL of ACN was added to the sample, and the mixture was agitated in a vortex (Reax Top, Heidolph, Germany) for 1 min. 

Then, 6.5 g of QuECheRS salts (catalog numbers 5982–6650) were added. The test tube was agitated in a vortex for 2 min and rotatory shaker for 15 min, and centrifuged for 5 min at 3154× *g* and 10 °C (Centrifuge 5810 R, Eppendorf AG 22331, Hamburg, Germany); 6 mL of the supernatant were recovered and transferred to a 15 mL test tube containing 1 g of the second batch of QuECheRS salts (catalog numbers 5982–5056). The tube was agitated in a vortex for 2 min and a rotatory shaker for 15 min, and centrifuged for 5 min at 3154× *g* and 10 °C. The obtained organic solution was filtered at 0.45 µm and transferred to a 1.8 mL vial for LC-MS/MS analysis.

### 2.7. Analytical Instrumentation

A UHPLC Agilent 1290 Infinity II LC coupled with a G7167B autosampler, a G7120A pump, and a GT116B MCT oven were connected to an Agilent 6470 Triple Quad LC/MS/MS mass detector with a MassHunter ChemStation. The column was a ZORBAX Eclipse Plus C18 (2.1 mm × 150 mm, 1.8 μm). The UHPLC analysis was conducted using a binary gradient: solvent A (H_2_O 5 mM in ammonium formate + 0.1% formic acid) and solvent B (methanol 5 mM ammonium formate + 0.1% formic acid). 

The elution gradient was T = 0, A 95%; T = 3.50 min, A 60%; T = 17 min, A 2%; and 10 min of post-run A 95%. The run’s total duration was 20 min, and retention times were 13.69 and 14.24 for fipronil and fipronil sulfone, respectively. The flow was 0.3 mL/min. 

The sample (2 μL) was injected in negative and positive modes. Mass detector conditions were gas and sheath-gas temperature 350 °C, gas flow 10 L/min, sheath-gas flow 12 L/min, nebulizer 30 psi, and capillary negative 3000 V. MRM transitions were m/z 435–330 and 435–250 for fipronil, 451–415 and 451–282 for fipronil sulfone.

### 2.8. Method Validation

An earlier validated multi-residue analytical method for detecting and quantifying 116 pesticides, including the MS/MS transitions of fipronil and fipronil sulfone, was adapted to eggs, feathers, muscle, and the internal organs (Table S1) [25,26]. The different components belonging to control animals were analyzed to ensure a total absence of the a.i.s. Selectivity was determined by comparing the control extract with blank samples spiked at the LOQ level. 

The absence of peaks at the active ingredients’ retention times was a criterion for confirmation of method selectivity. The linearity range was evaluated from a 6-point calibration curve that correlated the analytes’ peak area vs. concentrations. Linearity was acceptable when the coefficient of determination (r2) was >0.990. 

The detection limit (LOD) and the quantification limit (LOQ) were calculated three and ten times for the signal-to-noise ratio (S/N), considering the minimum concentration of the analyte in the spiked blank samples [27]. Recovery assays were conducted on eggs and feathers collected from control chickens, whereas poultry meat and organs were purchased from a local butcher shop. 

Recoveries data were reported as the mean of the recoveries obtained for all the matrices evaluated. Samples were spiked before extraction by adding an appropriate volume of mixed stock standard solution to reach LOQ, 10 × LOQ, and 100 × LOQ left for 30 min and processed according to the previous procedure. Recovery assays were conducted in triplicate for each matrix, and values were calculated as percentages vs. spiking concentration. 

Method precision (<20%) for eggs and feathers was expressed as repeatability (RSDr) using six replicates per day, whereas reproducibility (RSDwR) was calculated using two replicates per day for six working days; and both are expressed as the relative standard deviation (RSD). The matrix effect was evaluated by comparing the analytical responses of the pesticides dissolved in the eluent mixture and blank control matrix extracts. 

The expanded measurement uncertainty (U) was calculated by multiplying the combined uncertainty (u′) by a coverage factor, k = 2, at a 95% confidence level, using the equations:

u′ = √u′(𝑏𝑖𝑎𝑠)2 + u′(𝑝𝑟𝑒𝑐𝑖𝑠𝑖𝑜𝑛)2(1)

U = k × u′(2)

### 2.9. Chronic Dietary Exposure Assessments

Hazard quotient (HQ) was used to evaluate the risk for the long-term exposure of fipronil residues. HQ was calculated using the following formula:

HQ = EDI/ADI(3)

ADI (acceptable daily intake) was set at 0.0002 mg/kg bw per day [15]. EDI represents the estimated daily intake (mg/kg bw per day) and was calculated as follows:

EDI = F × C/bw(4)

F is the food consumption rate (kg/day); values of 0.018 (breast) and 0.026 (eggs) kg/day were used. Bw of 60 kg was used for adults. The concentration of fipronil residues (C) was expressed for the breast as the average value (mg/kg) obtained from the sum of fipronil and fipronil sulfone, and for the eggs as the average values of all sampling time in each trial (mg/kg) [28]. 

A value of HQ < 1 indicates that the lifetime consumption containing the measured level of pesticide residues should not pose health risks.

### 2.10. Acute Dietary Exposure Assessments

The estimation of the short-term intake (ESTI; mg/kg bw per day) was used to calculate the acute intake assessment with the following formula:

ESTI = F × C_h_/bw (5)

where C_h_ is the highest residue concentration of the sum of fipronil and fipronil sulfone obtained in breast and eggs after oral and dermal treatments [29].

The acute hazard quotient (aHQ) was calculated by the following equations:

aHQ = ESTI/ARfD (6)

The acute reference dose (ARfd; mg/kg bw per day) estimates the amount of a substance in food or drinking water that can be ingested over a short period, usually during one meal or one day, without appreciable health risk to the consumer. EFSA set the ARfd for fipronil at 0.009 mg/kg bw per day.

### 2.11. Transfer Factors (TFs)

To better explain the migration of residues of pesticides from feed to tissues, organs, or eggs, and their bio-distribution, transfers factors (TFs) obtained from dosing experiments are used. TFs for eggs, feathers, liver, kidney, muscle, heart, brain, and thigh obtained in trial 1 (oral) were calculated as follows:

TFs = Cs/Cf (7)

where Cs is the concentration of fipronil and fipronil sulfone founded in the different hen districts, and Cf is the concentration found in the polluted feed.

### 2.12. Statistical Analysis

Analysis of variance (ANOVA) was carried out with the software XLSTAT (Addinsolf LTD, Version 19.4). Mean comparisons of the effects of treatments were calculated by the Fisher’s least significant difference test at *p* ≤ 0.05.

## 3. Results

### 3.1. Formulate Composition

Analysis of the commercial fipronil formulations showed that all solutions were made by the parent compound (fipronil) and its primary metabolite, fipronil sulfone. Commercial products reported on their labels different starting concentrations of fipronil ranging from 100 mg/mL (B) to 2.50 mg/mL (A, F, and G) (Table 1). 

Fipronil/fipronil sulfone ratios were different in each formulation, with values of fipronil sulfone ranging from 3.95% (E) to 41.2% (A) and an average value among all the formulations of 14.10 ± 88.8 (% ± RSD) (Table 1). Therefore, formulation A was selected and used in trials 1 and 2 for feed contamination and dermal application on laying hens and roosters.

### 3.2. Method Validation

The LC-MS/MS LOQ values accounted for 0.51 µg/kg (s/n 999.77) and 0.10 µg/kg (s/n 28.52) for fipronil and fipronil sulfone (Table 2), respectively, in all matrices, which were below the MRL set by the actual legislation (5 µg/kg) [30]. 

The feed adopted is used in conventional agriculture, and the water was from a shallow well. All matrices were analyzed using a previously validated multi-residue method. Recovery assays conducted at three spiking levels showed apparent recoveries ranging from 90% to 110% with a precision (RSD%) from 3.7% to 8.4% for fipronil, and recoveries ranging from 83% to 102% with a precision (RSD%) from 6.3% to 13.0% for fipronil sulfone (Table 2). 

According to SANTE indications [31], the expanded uncertainty (U) was below 50% for both pesticides at all spiking levels (Table 2). The other validation parameters have been reported in the Supplementary Materials (Tables S2 and S3).

### 3.3. Eggs, Feathers, and Organs Contamination

#### 3.3.1. Oral Contamination (Trial 1)

Feed analysis repeated at T = 0 (after contamination) and T = 7 (last day of oral administration) showed an average concentration of 11.63 ± 2.91 mg/kg of fipronil and 14.02 ± 5.33 mg/kg of fipronil sulfone for a total amount of pesticide of 25.65 mg/kg (Table 3). After oral administration, fipronil was absorbed rapidly from the contaminated feed, with detectable residues in the eggs on the first day (Table 3). Eggs analysis showed that when the oral treatment was suspended on the seventh day (accounting for the higher fipronil residues), fipronil residues decreased continuously in the four successive weeks with a half-life time of 8 days. After one month, fipronil residues were undetectable (Table 3). Fipronil sulfone increased during the first week. Unlike fipronil, fipronil sulfone concentration was stable for almost one month, followed by a slight decrease in the second month (Table 3). This fact suggests a balance between the distribution and metabolism of the pesticide. 

The feather analysis showed the highest concentration of fipronil residues after one week, decreasing until the end of trial 1, in line with the eggs’ behavior. In contrast, fipronil sulfone levels continued to increase after two months (Table 3).

The laying hens showed higher amounts of fipronil in the thigh and the brain, whereas the liver and the breast showed average values near the LOQ of the method. No traces of fipronil were found in the heart muscle and the kidney (Table 3). 

Fipronil sulfone was found in all matrices. There were higher contamination levels in the heart muscle, liver, and kidney. In addition, the brain, thigh, and breast muscle showed values between 10 and 20 mg/kg (Table 3). 

At the end of the oral exposure experiment, fipronil sulfone showed wide bio-distribution in all muscles and internal organs. At the same time, the parent compound was found only in traces (Figure S1). 

#### 3.3.2. Dermal Contamination (Trial 2)

Dermal contamination by air spraying was performed only once at the beginning of trial 2. As a result, fipronil was never found in the eggs, internal organs, or muscles (Table 4, Figure S2). One day after the treatment, the average concentration of average fipronil sulfone in the eggs was 2.32 mg/kg, which increased to 279.13 mg/kg after 49 d and then decreased to 164.82 mg/kg after 56 d (hens ceased producing eggs) (Table 4).

Feathers analysis showed similar results in laying hens and roosters (Table 4). After 49 days, fipronil showed a decrease of 38.2%, and fipronil sulfone exhibited its maximum value (Table 4). 

After slaughtering, no fipronil was detected in any organ in either laying hens or roosters. Heart muscle, liver, kidney, and brain showed the highest levels of fipronil sulfone. In contrast, lower amounts were detected in the thigh and breast muscle, according to the oral administration results. Roasters showed similar results, although residues were slightly higher (Table 4).

#### 3.3.3. Non-Compliant Products

The eggs collected from farms subjected to confiscation of the non-compliant products had an average fipronil concentration of 0.0036 ± 12.67% mg/kg ± RSD% and an average fipronil sulfone concentration of 2.05 ± 13.09 mg/kg ± RSD% (Table 3).

### 3.4. Dietary Risk Assessment

The estimated daily intake (EDI) and the hazard quotient (HQ) calculated for eggs and breast showed values after the oral contamination trial of 0.056 and 0.003 mg/kg bw per day and 14.9 and 280.4, respectively, whereas after dermal exposure accounted for 0.035 and 0.016 mg/kg bw per day and 176.4 and 81.2, respectively (Table 5). 

The non-compliant eggs showed values for the EDI of 0.001 mg/kg bw per day and HQ of 4.11 (Table 5). 

The hazard quotients calculated for the acute dietary risk exposure (aHQ) in eggs and breast were 11.7 and 0.3 after oral exposure (trial 1) and 12.4 and 1.9 after dermal treatment (trial 2), respectively (Table 6). The aHQ obtained for non-compliant eggs accounted for 0.1 (Table 6).

## 4. Discussion

All the commercial fipronil formulations analyzed in this study showed the contemporary presence of fipronil and fipronil sulfone at different ratios, even if the labels reported only the parent compound (Table 1). Therefore, commercial formulation A was selected for the trials, having the highest fipronil/fipronil sulfone ratio, simulating the worst field scenario. 

Fipronil solutions can remain stable for about one year at room temperature in standard storage conditions [32]. However, rapid decomposition of fipronil in its metabolites occurs in the presence of metal ions or under alkaline conditions, indicating possible contamination of the formulations during the production chain [32]. Oral contamination studies have been reported on chicken fed with cyromazine premix (purity 99.8%) or pure fipronil in gelatine capsules [23,29] and on rats with atrazine administrated by oral gavage [33].

On the other hand, Canton et al. (2020) fed chickens with feed contaminated with a 1% commercial formulation of fipronil [13]. No residues were detected in chicken products obtained from the control group. Feed analysis showed a residues ratio of the a.i.s equivalent to the commercial fipronil solution composition (Table 3 and Table 4). 

After reaching the liver via the bloodstream, fipronil is extensively oxidized to fipronil sulfone by a cytochrome P-450 mediated process [6], resulting in this compound’s major route of hepatic metabolism [34]. In addition, the two compounds can go through enterohepatic recirculation from the gastrointestinal tract to the liver and vice versa, explaining the continuous decrease in fipronil and the increase in fipronil sulfone in eggs and feathers during the first month [35]. 

**Figure 1:**
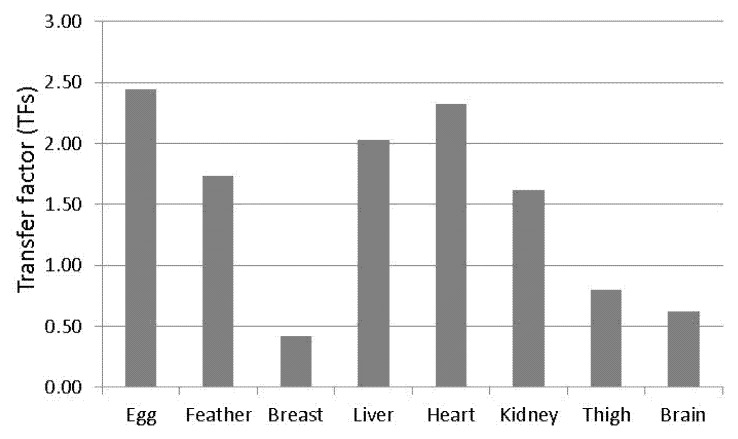
Fipronil and fipronil sulfone transfer factors (TFs) in different hen districts.

To better explain the migration of residues of pesticides from feed to tissues, organs, or eggs, and their bio-distribution, transfers factors (TFs) obtained from dosing experiments were used [29]. A TF is defined as the ratio of pesticide concentration in tissues or eggs to the concentration in the administered feed; this parameter is highly influenced by the affinity of the a.i.s for the different tissues, feeding levels, and feeding periods. In this study, TFs values decreased in the following order: egg > heart > liver > kidney > feather > thigh > brain > muscle (Figure 1) according to previous data obtained for fipronil (egg > liver) in chickens [36].

Fipronil and fipronil sulfone are lipophilic molecules (octanol/water partition coefficient = 4), and the yolk is a matrix with a high lipid content; thus, the two a.i.s easily migrate from feed to yolk. In addition, the datasheet of some pet formulations reported fipronil accumulation in fat and sebaceous glands, followed by a slow and sustained release of the pesticide for 30 days [37]. 

These data could explain the significant persistence of fipronil sulfone in the different districts of laying hens. After two doses of contaminated feed of fipronil, Canton et al. (2020) found fipronil and fipronil sulfone residues in eggs at 228.5 ± 79.8 µg/kg and 1.849 ± 867 µg/kg, respectively, considerably lower concentrations than those found in our study. However, the declared theoretical concentration of fipronil in the feed was 1 mg/kg, whereas in our study it was about 25 times higher and was administered for seven consecutive days [13]. 

Besides, the profile of growth and disappearance of the residues from eggs followed a similar trend for both fipronil and fipronil sulfone. Gerletti et al. (2020) reported a toxicokinetic model to predict the transfer of fipronil in eggs through the food chain [24]. However, their studies constituted repeated exposure trials (28 and 42 days) until reaching a steady state. Our study’s experimental data do not fit that model, indicating different behavior of fipronil after ingestion. Previous articles dealing with the bio-distribution of fipronil in rats after oral administration showed the highest levels in the liver and kidneys for males and ovaries for females [38]. 

Moreover, high levels of fipronil and its metabolites can alter the endocrine system and uterus development in rats [39]. Therefore, the high levels of fipronil sulfone accumulated in the animals’ organs in trial 2 and trial 1 can explain the atrophy of the uterus after 56 days.

The higher fipronil sulfone concentration in roosters’ organs depends on the structure of their feathers. The so-called “developing feathers,” typical of young specimens in the growth step, shows a more developed blood circulation in the calamus and follicles than feathers belonging to adults, allowing an increase in the diffusion of the pesticide into the body [40]. 

Fipronil shows high susceptibility to environmental conditions [34]. When exposed directly to air and light, it can follow two metabolic routes, oxidation and photolysis, leading to the formation of fipronil sulfone and fipronil-desulfinyl, respectively [41]. Therefore, the lack of fipronil in eggs and organs after dermal contamination could be explained by different factors: a degradation rate of fipronil-to-fipronil sulfone higher than the transfer rate from the feathers to the body, or extensive exogenous oxidation of the remaining fipronil after application. 

Furthermore, data in the literature have shown that pollutants can concentrate in the lipidic preen oil glands and feathers, and contamination from microorganisms and environmental pollutants can occur in birds during preening activities (10% of bird activity) [42]. Therefore, researchers have assumed that fipronil can enter the organism through preening after dermal contamination. However, our data showed that, when dermal contamination occurred, no fipronil residues were detected in the eggs or any organ of the hens (trial 2).

After oral ingestion, all organs and feathers were contaminated by fipronil and its metabolite (trial 1). Thus, in our trial, fipronil sulfone entered the body through the feathers’ blood vessels and not from ingestion during preening. 

Fipronil’s and fipronil sulfone’s behaviors in chickens after dermal pollution have not been extensively studied. EFSA reported low dermal absorption by rat and human skin, but no data have been reported for contaminated feathers [43]. After a single dermal exposure trial on laying hens, depositing two drops of fipronil solution on the caudal half of the animal’s dorsal line, Canton et al. (2020) found traces of fipronil in eggs for ten days. Fipronil sulfone was detectable in all the trials at a maximum residue value of 163 ± 26 µg/kg, much lower than data obtained in our study [13]. This discrepancy could depend on the different concentrations administered in the two studies and the different method of dermal application. 

Data obtained in trials 1 and 2 for fipronil sulfone residues in eggs showed that the dermal route promotes slower accumulation of the a.i.s than oral administration. However, topical administration led to final concentrations of fipronil sulfone in the internal organs and the muscle about ten times higher those resulting from oral administration. 

This phenomenon can be explained by the high concentration of the spray product applied during trial 2 associated with avoiding the first hepatic passage. In addition, the selected fipronil formulation is composed of about 10% ethanol and various emulsifiers, well known for their transdermal enhancer properties [44]. 

Two months after treatments, both fipronil and fipronil sulfone were still detectable in feathers after oral and dermal administration. In addition, as suggested by data obtained in trial 1, when fipronil residues were found in the eggs, recent ingestion of the parent pesticide by laying hens may have occurred. After that, the contemporary presence of fipronil sulfone in feathers and eggs associated with the absence of fipronil in eggs could be a symptom of dermal contamination or old oral contamination. 

Monitoring trials, biomonitoring, and inspection control analysis of poultry are conducted on meat and lipids samples after slaughtering. However, by evaluating the residues of the two a.i.s in feathers and eggs, it might be possible to create a cross-monitoring system to avoid sacrificing the animals and better explain the origins of egg and poultry contamination in breeding farms. 

Various authors have reported on methods to determine environmental pollution or accidental pesticide contamination by directly analyzing the feathers of live birds without slaughtering [45], increasing animal welfare, as stated previously. However, biomonitoring plans that include feathers should consider purposes, selection (body or flight feathers), treatment, and storage [45].

Moreover, the relationship between feather concentrations and meat/egg concentrations must be well established before risk assessment studies. After that, further evaluations in subsequent studies must be considered.

Information on acute and chronic exposure to fipronil in the human population is limited. However, fipronil has neurotoxic, hepatotoxic, nephrotoxic, and cytotoxic effects on humans and animals [46]. Moreover, several studies have dealt with the relationship between fipronil and severe thyroid disease [47]. 

Based on data showing an increase in thyroid follicular cell tumors in rats after long term exposure, the US Environmental Protection Agency (EPA) has classified fipronil as a possible human carcinogen. Although the modes of action of fipronil and its metabolites are relatively unknown, oxidative stress has been suggested to play a critical role in fipronil-induced toxicities. They may alter the antioxidant defense system, thereby damaging cellular macromolecules such as lipids, proteins, and DNA.

All the egg and muscle samples analyzed and used for the dietary risk assessment had values higher than the MRL set for poultry products (0.005 mg/kg). In addition, the high concentrations of fipronil sulfone found in all districts of the hens after the trials coupled with low values of ADI and ARfD set by EFSA for fipronil led to high values of aHQ and HQ in all cases. 

Regarding chronic exposure, the worst scenarios were represented by the eggs collected from trials 1 and 2 (in that order; Table 5), followed by the muscle obtained after trial 2 and trial 1 (in that order). HQ value of non-compliant eggs was 4.11 times higher than the value not presenting health risks (HQ < 1) (Table 5). 

In general, the acute hazard quotients were lower than the chronic quotients. Values for the muscle obtained from trial 1 and the non-compliant eggs were less than 1 and were the only poultry products safe for human health in this study (Table 6). Different authors have performed dietary risk assessments on eggs and muscles contaminated by fipronil [48].

## 5. Conclusions

This study investigated fipronil and fipronil sulfone bio-distribution after the simulation of incorrect oral and dermal administration in laying hens and roosters in breeding farms. Furthermore, chronic and acute dietary risk assessments were also performed to understand better the impact that high contaminated poultry products could have on human health. 

Our data showed that the contamination of laying hens and roosters after oral ingestion or dermal contact followed different routes. When oral administration occurred, fipronil and its primary metabolite were found in all parts of the body—eggs, meat, various organs, and feathers. When contamination was from dermal spraying, fipronil sulfone was detected inside the body and in feathers, whereas fipronil was detected only in feathers and was utterly absent in the eggs. Thus, the contemporary presence of fipronil sulfone in feathers and eggs associated with the lack of fipronil in eggs might suggest dermal contamination or previous oral contamination. 

Data obtained for HQ and aHQ after dietary risk assessment confirmed that incorrect use of unauthorized pesticides could lead to severe contamination of entire poultry farms with consequent risks to both animal health, and through the food chain, human health. Therefore, education campaigns should be encouraged to inform farmers about the health-related risks of using this pesticide on hens. 

Moreover, monitoring studies and inspections surveys on animals should use the most suitable and ethical method for the animals’ wellness. The data reported in this paper highlighted that animals do not need to be killed because the analysis of feathers detects fipronil and fipronil sulfone up to 2 months after contamination. This approach can increase the number of hens, pullets, and roosters—though the latter are usually ignored—available for analysis in a community, leading to the creation of large-scale monitoring plans.

## Figures and Tables

**Table 1:** Commercial formulations’ chemical compositions investigated by UHPLC MS/MS.

Formulate (mg/mL)	Fipronil (mg/mL ± RSD%)	Fipronil Sulfone (mg/mL ± RSD%)	Fipronil Sulfone (%)
A (2.50)	1.04 ± 5.21	1.03 ± 6.14	41.2
B (100)	93 ± 3.15	4.42 ± 4.56	7.32
C (67.70)	82 ± 2.56	9.43 ± 2.70	15.46
D (61.00)	64 ± 2.49	4.72 ± 3.02	7.74
E (61.00)	59 ± 3.08	2.40 ± 5.09	3.95
F (2.50)	2.19 ± 2.15	0.30 ± 7.15	12.0
G (2.50)	2.20 ± 2.03	0.27 ± 9.16	11.0

**Table 2:** LOD, LOQ, recoveries, and expanded uncertainty of fipronil and fipronil sulfone spiked at different concentrations.

Cpd Name	LOD (µg/kg)	LOQ (µg/kg)	Recovery * (Mean ± RSD%) and Expanded Uncertainty (%)					
			LOQ	U (%)	10 × LOQ	U (%)	100 × LOQ	U (%)
Fipronil	0.10	0.51	110 ± 8.4	34.18	90 ± 3.7	28.87	99 ± 5.4	23.17
Fipronil sulfone	0.05	0.10	83 ± 13.0	46.92	98 ± 6.3	24.19	102 ± 9.4	28.36

* Mean ± RSD% of the recoveries obtained for eggs, feathers, muscle, and internal organs.

**Table 3:** Concentrations of fipronil and fipronil sulfone (mg/kg) in the samples from the feeding experiment (trial 1).

Day	Eggs		Feathers	
	Fipronil	Fipronil Sulfone	Fipronil	Fipronil Sulfone
0 *	<0.001	<0.001		
1 *	0.18 (a)	0.21 (a)		
2	0.16 (a)	0.19 (a)		
7 ^y^	0.80 (b)	237.32 (b)	2.92 (a)	10.40 (a)
14	0.43 (c)	248.40 (b)	1.36 (a)	16.33 (a, b)
21	0.29 (a)	262.27 (b)		
28	0.11 (a)	225.89 (b)		
35	<0.001	228.59 (b)		
42	<0.001	197.49 (c)	0.99 (b)	20.03 (b)
49	<0.001	126.42 (d)		
56	<0.001	118.12 (d)		
63	<0.001	111.25 (d)		
66	<0.001	62.68 (e)	0.25 (c)	44.33 (c)
Feed	11.63 ± 2.91	14.07 ± 5.33		
Liver	0.006 (a)	52.17 (a)		
Heart	<LOQ	59.66 (a)		
Kidney	<LOQ	41.59 (b)		
Thigh	0.083 (b)	20.52 (c)		
Breast	0.010 (a)	10.74 (d)		
Brain	0.066 (b)	15.95 (c, d)		
Non-compliant	0.0036 ± 12.67	2.05 ± 13.09		

* Day of the start of feeding contamination treatment. ^y^ End of feeding contamination. Values among columns (in brackets) followed by unalike letters differ significantly by Fisher’s least significant difference (LSD) procedure, *p* ≤ 0.05.

**Table 4:** Concentrations of fipronil and fipronil sulfone (mg/kg) in the samples from the air spraying (trial 2).

Day	Laying Hens				Roaster	
	Eggs		Feathers		Feathers	
	Fipronil	Fipronil sulfone	Fipronil	Fipronil sulfone	Fipronil	Fipronil sulfone
0 *	<0.001	<0.001				
1	<0.001	2.32 (a)	195.85 (a)a	74.71 (a)a	166.66 (a)a	71.73 (a)a
7	<0.001	16.26 (b)	166.97 (a)a	337.94 (b)a	132.97 (a)a	324.35 (b)a
14	<0.001	27.89 (b)				
21	<0.001	44.12 (c)				
28	<0.001	46.85 (c)				
35	<0.001	131.55 (d)				
42	<0.001	169.27 (e)				
49	<0.001	279.13 (f)	120.88 (b)a	540.21 (c)a	113.06 (b)a	531.79 (c)a
56 ^y^	<0.001	164.82 (e)				
63						
66			60.34 (c)a	142.49 (d)a	79.12 (c)b	191.45 (d)b
Spray S.	1131.53 ± 8.91	1368.75 ± 9.12				
Liver	<0.001	208.69 (a)a			<0.001	303.25 (a)b
Heart	<0.001	278.34 (a)a			<0.001	506.94 (b)b
Kidney	<0.001	192.70 (a)a			<0.001	307.93 (a)b
Thigh	<0.001	91.02 (b)a			<0.001	110.22 (c)a
Breast	<0.001	58.65 (c)a			<0.001	70.47 (d)a
Brain	<0.001	106.32 (b)a			<0.001	105.47 (c)a

* Day of spray treatment. ^y^ End of egg-laying. Values among columns (in brackets) followed by unalike letters differ significantly by Fisher’s least significant difference (LSD) procedure, *p* ≤ 0.05. Values among row (without brackets) followed by unalike letters differ significantly by Fisher’s least significant difference (LSD) procedure, *p* ≤ 0.05.

**Table 5:** Chronic dietary risk assessment parameters.

Matrix	MRL (mg/kg)	F (kg/day)	ADI (mg/kg bw per day)	C (mg/kg)	EDI (mg/kg bw per day)	HQ
Breast	0.005	0.018	0.0002	10.7 (trial 1) 58.6 (trial 2)	0.003 (trial 1) 0.016 (trial 2)	14.9 (trial 1) 81.2 (trial 2)
Eggs	0.005	0.026	0.0002	140.2 (trial 1) 88.2 (trial 2)	0.056 (trial 1) 0.035 (trial 2)	280.4 (trial 1) 176.4 (trial 2)
Non-compliant eggs	0.005	0.026	0.0002	2.05	0.001	4.11

MRL: maximum residue limit; F: food consumption rate; ADI: acceptable daily intake; C: fipronil concentration (sum of fipronil and fipronil sulfone); EDI: estimated daily intake; HQ: hazard quotient.

**Table 6:** Acute dietary Risk assessment parameters.

Matrix	MRL (mg/kg)	F (kg/day)	ARfD (mg/kg bw per day)	C (mg/kg)	ESTI (mg/kg bw per day)	aHQ
Breast	0.005	0.018	0.009	10.92 (trial 1) 62.60 (trial 2)	0.003 (trial 1) 0.017 (trial 2)	0.3 (trial 1) 1.9 (trial 2)
Eggs	0.005	0.026	0.009	262.56 (trial 1) 279.13 (trial 2)	0.105 (trial 1) 0.112 (trial 2)	11.7 (trial 1) 12.4 (trial 2)
Non-compliant eggs	0.005	0.026	0.009	2.32	0.001	0.10

MRL: maximum residue limit; F: food consumption rate; ARfD: acute reference dose; C: fipronil concentration (sum of fipronil and fipronil sulfone); ESTI: estimation of the short-term intake; aHQ: acute hazard quotient.

## Supplementary Materials

The following are available online at https://www.mdpi.com/article/10.3390/foods10123077/s1. Table S1: Active ingredients investigated in the multiresidue UHPLC MS/MS method, Table S2: Regression equation and r^2^ of the active ingredients, Table S3: Intra-day repeatability and inter-day reproducibility of fipronil and fipronil sulfone concentrations in eggs and feathers, Figure S1: MRM chromatogram of fipronil and fipronil sulfone in egg sample after oral exposure, Figure S2: MRM chromatogram of fipronil and fipronil sulfone in feathers samples after oral (A) and dermal (B) exposure.
