# Supplementary material for: Fipronil and Fipronil Sulfone Distribution in Chicken Feathers and Eggs after Oral and Dermal Exposure
Source: Foods. 2021 Dec 10;10(12):3077. doi: 10.3390/foods10123077 (PMC8702044; doi:10.3390/foods10123077)
Supplement: Supplementary file 1 [file foods-10-03077-s001.zip › foods-1456194-supplementary.pdf]

Supplementary Materials

Table S1. Active ingredients investigated in the multiresidue UHPLC MS/MS method.

Cpd Name	MRM*	r.t.	Cpd Name	MRM	r.t.	Cpd Name	MRM	r.t.
Methamidophos	142>94	2.84	Fenamidone	312>92	12.04	Difenconazole	406>251	14.99
Cyromazine	167>85	2.98	Boscalid	343>307	12.19	Chlorpyrifos-methyl	321>125	15.02
Formetanate	222>165	3.30	Mandipropamid	412>328	12.24	Ametoctradin	276>149	15.07
Acephate	184>143	3.49	Dimethomorph	388>301	12.43	Trifloxystrobin	409>186	15.18
Propamocarb	189>102	3.60	cyproconazole	292>70	12.50	Cycloate	216>83	15.20
Pymetrozine	218>105	3.60	Benthiavalicarb	340>180	12.67	Indoxacarb	528>203	15.22
Omethoate	214>125	3.90	Molinate	188>126	12.77	Hexaflumuron	461>158	15.24
Oxamyl	237>72	4.44	Chloroxuron	291>72	12.79	Cycloxydim	326>180	15.70
Carbendazim	192>160	4.83	Spirotetramat	374>302	12,80	Quizalofop-ethyl	373>271	15.76
Methomyl	163>88	4.89	Myclobutanil	289>125	12.86	Emamectin Benzoate	887>158	15.76
Thiamethoxam	292>211	4.96	Bifenazate	301>198	12.91	Buprofezin	306>201	15.89
Monocrotophos	224>127	5.11	Triadimenol	296>70	12.99	Metaflumizone	507>178	15.94
Chlordimeform	197>117	5.13	Azinphos-ethyl	346>97	13,09	Tebufenpyrad	334>117	15.96
Imidacloprid	256>175	5.74	Fenhexamid	302>97	13.11	Propaquizafop	444>299	16.03
Methiocarb	242>185	5.86	Mepanipyrim	224>106	13.18	Allethrin	303>135	16.10
Acetamiprid	223>126	6.10	Tetraconazole	372>159	13.21	Lufenuron	510>158	16.15
Dimethoate	230>125	6.19	Bupirimate	317>166	13.22	Oxadiazon	362>220	16.16
Cymoxanil	199>128	6.64	Flufenacet	364>142	13.24	Piperonyl butoxide	356>177	16.24
Thiacloprid	253>126	6.82	Iprovalicarb	321>119	12.95	Pyriproxyfen	322>96	16.34
Atrazine-desethyl	188>146	6.92	Cyazofamid	325>108	13.27	Chlorpyriphos	350>198	16.41
Aldicarb	213>89	7.43	Napropamide	272>58	13.36	Hexythiazox	353>228	16.45
Pirimicarb	239>72	8.02	Fipronil	435>250	13.69	Pendimethalin	282>212	16.55
Dichlorvos	221>109	8.60	Cyprodinil	226>77	13.59	Flufenoxuron	489>158	16.62
Thiophanate-methyl	343>151	8.61	Fenamiphos	304>217	13.64	Propargite	368>231	16.71
Metribuzin	215>187	8.70	Flusilazol	316>165	13.67	Etoxazole	160>141	16.74

Carbofuran	222>165	8.77	Iprodione	330>245	13.78	Fenpyroximate (E)	422>366	16.99
Carbaryl	202>145	9.30	Fipronil sulfone	451>282	14.24	Deltamethrin	523>281	17.16
Imazalil	297>159	9.84	Aclonifen	265>182	13.96	Acrinathrin	559>208	17.32
Fosthiazate	284>61	9.94	Penconazole	284>70	13.96	Pyridaben	365>147	17.48
Disulfoton-Sulfoxide	291>185	10.10	Tebuconazole	308>70	14.19	T-Fluvalinate	503>208	17.51
Flutriafol	302>95	10.38	Benalaxyl	326>148	14.34	Bifenthrin	442>181	18.20
Metalaxyl	280>160	10.88	Spinosyn A	732>142	14.42	Fenarimol	331>313	18.13
Pyrimethanil	200>106	10.88	Zoxamide	336>187	14.45	Etofenprox	394>177	18.21
Methidathion	303>145	11.00	Bitertanol	338>70	14.62			
Azinphos-methyl	318>261	11.23	Pyraclostrobin	388>194	14.63			
Chlorantraniliprole	483>285	11.39	Cyflufenamid	413>295	14.67			
Azoxystrobin	404>344	11.73	Clofentezin	303>138	14.69			
Diethofencarb	268>226	11.78	Phosalone	368>182	14.74			
Propanil	218>162	11.92	Metrafenone	409>209	14.88			
diclobutrazol	328>70	11.99	Spinosyn D	746>142	14.99			

*Mass transition used for quantitative analysis.

Table S2. Regression equation and r^2 of the active ingredients.

	Fipronil	Fipronil sulfone
	0.5-517 μg/L	0.1-1031 μg/L
Regression equation	Y=157894.21x + 3031.09	Y=11400.78x + 140.94
r^2	0.9929*± 0.40%	0.9998 ± 0.85%

* 6-point calibration curve (three replicates).

Table S3. Intra-day repeatability and inter-day reproducibility of fipronil and fipronil sulfone concentrations in eggs, and feathers.

Standard conc.	precision RSD%			
(mg/kg)	Intra-day (n=6)		Inter-day (n=12)	
Fipronil	r. time	area	r. time	area
		eggs		
0.51	0.09	17.27	0.07	17.94
1.02	0.07	12.42	0.08	18.22
10.2	0.07	6.34	0.07	12.69
		feathers		
0.51	0.06	6.52	0.05	9.51
1.02	0.08	7.16	0.07	10.02
10.2	0.05	5.49	0.05	7.98
Fipronil sulfone				
		eggs		
0.10	0.06	2.64	0.07	7.13
1.01	0.05	10.13	0.05	14.13
10.1	0.06	9.74	0.06	11.12
		feathers		
0.05	0.05	5.69	0.09	6.56
1.01	0.07	5.61	0.09	8.45
10.1	0.05	6.45	0.06	7.56

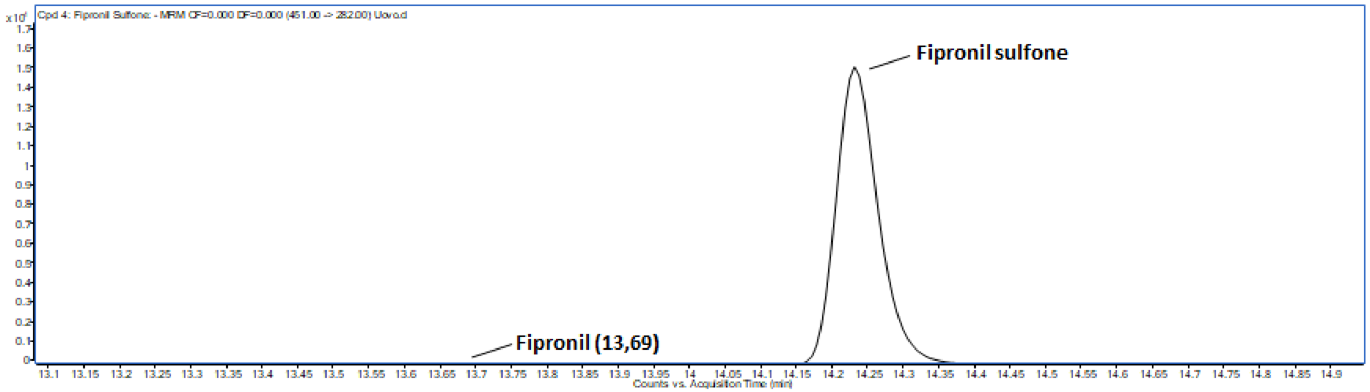

Figure S1: MRM chromatogram of fipronil and fipronil sulfone in egg sample after oral exposure.

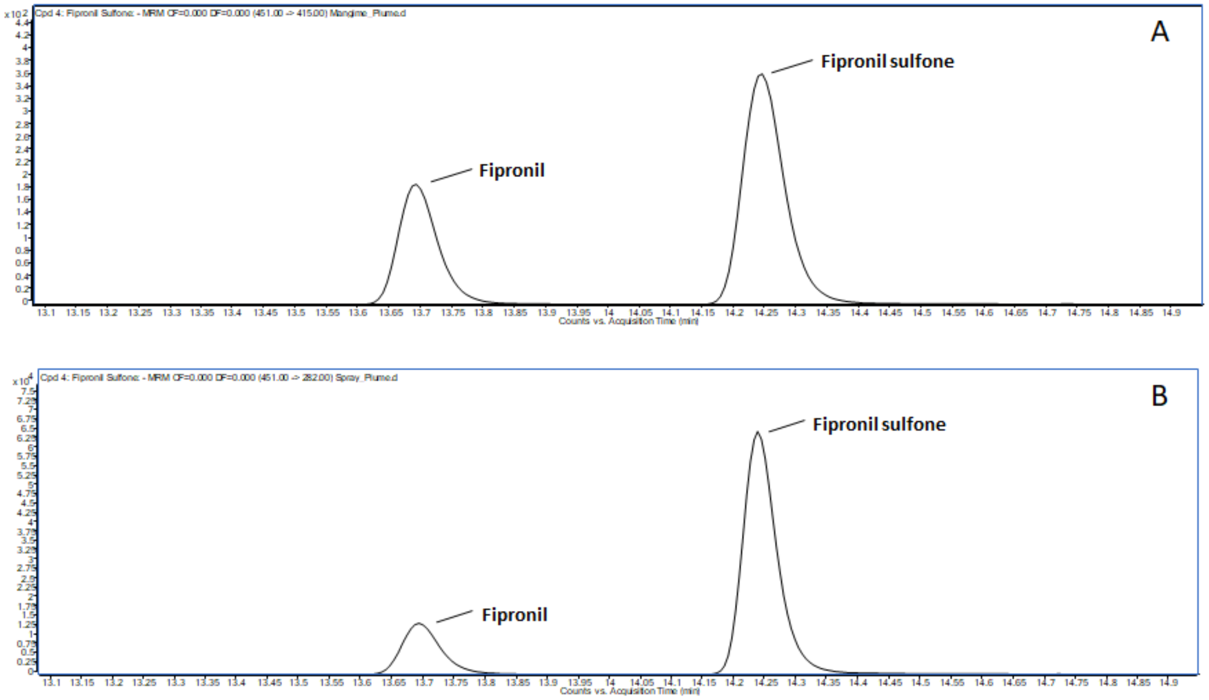

Figure S2: MRM chromatogram of fipronil and fipronil sulfone in feathers samples after oral (A) and dermal (B) exposure.
